# Regulation of Neutrophil Senescence by MicroRNAs

**DOI:** 10.1371/journal.pone.0015810

**Published:** 2011-01-19

**Authors:** Jon R. Ward, Paul R. Heath, James W. Catto, Moira K. B. Whyte, Marta Milo, Stephen A. Renshaw

**Affiliations:** 1 MRC Centre for Developmental and Biomedical Genetics, School of Medicine and Biomedical Science, The University of Sheffield, Sheffield, United Kingdom; 2 Department of Infection and Immunity, School of Medicine and Biomedical Science, The University of Sheffield, Sheffield, United Kingdom; 3 Department of Neuroscience, School of Medicine and Biomedical Science, The University of Sheffield, Sheffield, United Kingdom; 4 NIHR Cardiovascular Biomedical Research Unit, School of Medicine and Biomedical Science, The University of Sheffield, Sheffield, United Kingdom; 5 Department of Oncology, School of Medicine and Biomedical Science, The University of Sheffield, Sheffield, United Kingdom; National Institute of Allergy and Infectious Diseases, National Institutes of Health, United States of America

## Abstract

Neutrophils are rapidly recruited to sites of tissue injury or infection, where they protect against invading pathogens. Neutrophil functions are limited by a process of neutrophil senescence, which renders the cells unable to respond to chemoattractants, carry out respiratory burst, or degranulate. In parallel, aged neutrophils also undergo spontaneous apoptosis, which can be delayed by factors such as GMCSF. This is then followed by their subsequent removal by phagocytic cells such as macrophages, thereby preventing unwanted inflammation and tissue damage. Neutrophils translate mRNA to make new proteins that are important in maintaining functional longevity. We therefore hypothesised that neutrophil functions and lifespan might be regulated by microRNAs expressed within human neutrophils. Total RNA from highly purified neutrophils was prepared and subjected to microarray analysis using the Agilent human miRNA microarray V3. We found human neutrophils expressed a selected repertoire of 148 microRNAs and that 6 of these were significantly upregulated after a period of 4 hours in culture, at a time when the contribution of apoptosis is negligible. A list of predicted targets for these 6 microRNAs was generated from http://mirecords.biolead.org and compared to mRNA species downregulated over time, revealing 83 genes targeted by at least 2 out of the 6 regulated microRNAs. Pathway analysis of genes containing binding sites for these microRNAs identified the following pathways: chemokine and cytokine signalling, Ras pathway, and regulation of the actin cytoskeleton. Our data suggest that microRNAs may play a role in the regulation of neutrophil senescence and further suggest that manipulation of microRNAs might represent an area of future therapeutic interest for the treatment of inflammatory disease.

## Introduction

Neutrophils are the most abundant white blood cell in the body, playing an essential role in the destruction of invading bacterial and fungal pathogens. They are rapidly recruited to sites of injury, where they extravasate into the tissues and destroy the pathogen via several mechanisms, including phagocytosis, and the release of antimicrobial substances [1]. Activated neutrophils also release a plethora of proinflammatory mediators such as IL-8, IP-10 (CXCL-10) [2] and leukotriene B4 [3], recruiting and activating further inflammatory cells, thus enhancing the inflammatory response.

Neutrophil functions are limited by a process of neutrophil senescence, which renders neutrophils unable to respond to chemoattractants, carry out respiratory burst, or to degranulate [4], [5]. Senescence is accompanied by an upregulation of CXCR4 on the neutrophil surface and a corresponding increase in ability to migrate to SDF-1 [6]; these changes home neutrophils back to the bone marrow where they undergo apoptosis [7]. In parallel, aged neutrophils can also undergo spontaneous apoptosis, with a half-life of less than 8 hours [8]. This is followed by their subsequent removal by phagocytic cells such as macrophages, a mechanism to prevent unwanted inflammation and tissue damage [9]. GMCSF is a cytokine with multiple effects on neutrophil maturation, and on the function of inflammatory neutrophils. The actions of GMCSF include delaying neutrophil senescence, in part by the suppression of neutrophil apoptosis [10], [11]. Neutrophil apoptosis is thought to be a major factor in the functional senescence of neutrophils [11], and the role of changes in protein expression caused by microRNAs have not to date been investigated.

The mechanisms of the regulation of neutrophil senescence have not been well characterised. It is now well known, however, that neutrophil lifespan is exquisitely regulated both positively and negatively by interaction with environmental cues such as bacterial products or cytokines. Rates of neutrophil apoptosis can be increased by the ingestion of pathogens such as *E.coli* [12], and through ligation of cell surface receptors such as Fas [13] and TRAIL-R2 (DR5) [14]. Conversely, the lifespan of a neutrophil can be extended through stimulation by inflammatory mediators such as GMCSF and TNFα[10], with associated functional longevity of pro-inflammatory and anti-microbial functions [11]. New protein translation is important in maintaining neutrophil functional viability: blocking either translation or transcription increases the rate of neutrophil apoptosis [15], [16]. We therefore hypothesised that dynamic changes in microRNA levels within neutrophils might regulate proteins instrumental in determining neutrophil functional longevity.

The pattern of proteins expressed within a cell is thought to be fine tuned by expression of large numbers of microRNAs, a recently discovered family of short RNA species that regulate gene expression using the RNA interference pathway [17]. The immune system is regulated at many levels by microRNA action, including differentiation and proliferation of myeloid lineages, and cellular responses to proinflammatory stimuli [18]. Several recent studies have reported the presence of microRNAs within human neutrophils [19], [20], [21], [22], yet their role in defining neutrophil functions has not been reported. Mature microRNAs are 20–25 nucleotides in length and are produced from a primary transcript (pri-miRNA), usually several kilobases long, which is cleaved in the nucleus by Drosha and its cofactor DGCR8 [23] to leave a pre-miRNA of about 70bp. Pre-miRNA is then transported into the cytosol by exportin 5 [24] followed by cleavage by Dicer to leave mature microRNA [25]. There are currently over 850 human microRNAs (www.miRBase.org V12) and they are thought to regulate up to 30% of the human genome [26]. Due to incomplete basepairing with the target sequence, a single microRNA can target many hundreds of genes, and a single gene can be regulated by multiple microRNAs, allowing fine tuning of gene transcription [27]. Key determinants of neutrophil functional longevity, such as Mcl-1, are known to be regulated by microRNAs in other cell types [28], [29]. We therefore examined changes in microRNA expression over time and upon treatment with GMCSF in human neutrophils, revealing for the first time the co-ordinated dynamic regulation of microRNAs, and hence a range of target genes with roles in regulation of neutrophil function. This work begins to identify how neutrophils use microRNAs as one of the tools available to limit the proinflammatory potential of these essential, but potentially harmful host defence cells.

## Materials and Methods

### Neutrophil preparation

Peripheral venous blood was taken with informed consent using protocols approved by South Sheffield Research Ethics Committee. South Sheffield Research Ethics Committee specifically approved this study. All donors gave written consent. Following dextran sedimentation, Peripheral Blood Neutrophils (PMNs) were prepared by density centrifugation using Optiprep™ (Axis-Shield, Upton Huntingdon, UK) [30]. Briefly, leukocyte rich plasma was resuspended in Hanks' buffered salt solution (HBSS)+20% platelet poor plasma (PPP) and Optiprep™ to give a density of 1.13 g/l. Optiprep™ /HBSS+20% PPP was also prepared at 1.095 and 1.080 g/l and layered over the top of the cell suspension, before a further layer of HBSS+20% PPP was added. Cells were then centrifuged at 700×*g* for 30 minutes before harvesting of the PMN layer from the 1.095/1.080 interface and further purification by negative magnetic selection using a custom cocktail mix (Stemcell Technologies, Vancouver, Canada) [31]. Neutrophils were then left in culture media for 1 and 4 hours with or without GMCSF (50U/ml) (Sigma-Aldrich, Poole, UK), followed by preparation of total RNA. To assess neutrophil purity, duplicate cytospins were prepared at time 0, and 300 cells per slide were counted as either neutrophil or non-neutrophil depending on their morphology. Rates of neutrophil apoptosis were determined through preparation of cytospins at 20 hours. Duplicate slides were counted blind, and 300 cells per slide were assessed as either apoptotic or non-apoptotic neutrophils.

### RNA preparation

Total RNA was prepared from 3–5×10^6^ neutrophils using the *mir*Vana™ miRNA isolation kit (Ambion, Huntingdon, UK) enabling the purification of small RNA species as well. RNA concentration, 260/280 and 260/230 values were assessed using a NanoDrop® (NanoDrop, Wilmington, DE, USA), and analysis of RNA integrity was performed using the Agilent 2100 RNA Bioanalyser (Agilent Technologies, Wokingham, UK) using the Nano 6000 kit (Agilent Technologies).

### MicroRNA microarray and data analysis

A custom microRNA microarray was designed using eArray (Agilent Technologies) comprising of 2371 different probes for 851 human microRNAs (Agilent Human miRNA microarray V3, Sanger Database V12). Microarrays were of the 8×15K format, giving 16 replicates for each individual microRNA in each sample. One hundred nanograms of total RNA was labelled with Cyanine 3-pCp and hybridised to the chip according to the manufacturer's instructions. Following careful washing, the arrays were read using the Agilent microarray scanner and the data were extracted using Feature Extraction V10.7 (Agilent Technologies).

Extracted data were transferred into Microsoft Excel (Microsoft Corporation, Reading, UK) where all data were transformed to Log base 2. Transformed data were then corrected within each chip using the median percentile shift to 5 abundantly expressed positive control probes on each array. Data were then further normalised using the median percentile shift to 2 microRNAs that were identified to be unregulated across samples using NormFinder [32]. The microRNAs identified were miR-16, which has been previously described as suitable for normalisation in a RT-PCR study of human breast cancer tissue [33] and miR-720. Fold change was then simply calculated through the subtraction of one value from another. Where direct comparisons were performed, each sample was within the same chip.

### Real time PCR

Real time PCR was carried out on total RNA prepared using the *mir*Vana™ miRNA isolation kit as described above using 3 different neutrophil donors. The Taqman® microRNA reverse transcription kit (Applied Biosystems, Warrington, UK) and Taqman® microRNA assays (Applied Biosystems) were used according to the manufacturer's instructions and plates were read using an ABI Prism 7900 Real-Time PCR system (Applied Biosystems).

### Analysis of predicted microRNA binding sites

A list of genes with predicted binding sites for the selected microRNAs was downloaded from http://mirecords.biolead.org/. Only those genes that were predicted by at least 3 of the algorithms were selected. Data were imported into Microsoft Excel and the target genes were compared with those genes reported to be downregulated by at least two-fold after 3 or 6 hours in culture [34]. Those downregulated genes with binding sites for at least 2 out of the 6 selected microRNAs were then subjected to pathway analysis at http://david.abcc.ncifcrf.gov/tools.jsp [35].

## Results

### Human neutrophils express a selected repertoire of microRNAs

**Figure 1:**
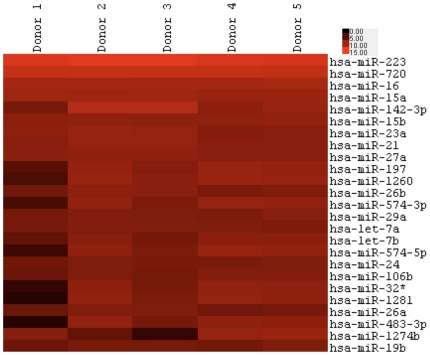
Freshly isolated human neutrophils express a discrete repertoire of microRNAs. Total RNA from freshly isolated human neutrophils (purity 97.9%±0.21; apoptosis rates at 20 hours 62.4%±7.4; n = 5) were analysed for microRNA expression by microarray. In total 148 microRNAs were found to be present (as indicated by the Feature Extraction software) in at least 4 out 5 donors. The heat map shows the 25 most abundant microRNAs in freshly isolated human neutrophils.

To determine the microRNA content of human neutrophils we carried out a one-colour custom 8×15K microarray (Agilent Technologies) designed to robustly detect all known microRNAs. Before preparation of microRNA samples, the purity of the enriched human neutrophils was assessed by cytospin to be 97.9%±0.21 (n = 5, individual purities 98, 97.15, 97.95, 98.25, 98), with the very low level of contaminating cells identified as eosinophils. Elimination of monocyte contamination is critical in these experiments, since monocytes have been shown to modulate neutrophil responses to inflammatory stimuli [36] and may theoretically contribute disproportionately to the detectable microRNA population. In contrast, eosinophil contamination has little functional effect on neutrophils [31], and is unlikely to contribute significantly to the microRNA profiles seen. However, a detailed analysis of eosinophil miRNAs has not been published, and the possibility that some of the findings may be due to eosinophil contamination cannot be completely excluded. Apoptosis rates of the neutrophils at 20 hrs were also assessed by cytospin and determined to be 62.4%±7.4 (n = 5), which is within the range seen in our laboratory and indicative of low levels of neutrophil activation [37]. Following preparation of sufficiently high quality total RNA, samples were labelled, loaded onto the array slides and read following the manufacturer's instructions. The data were extracted using Feature Extraction V10.7 (Agilent Technologies) and data were transferred into Microsoft Excel for analysis. In total, 146 out of the 851 microRNAs were found to be present in at least 4 out of 5 donors, as indicated by the Feature Extraction software at time 0, with this value increasing to 190 if the threshold was lowered to 3 out of 5 donors. Figure 1 shows a heat map of the 25 most abundant microRNAs in 5 human neutrophil donors. Table S1 shows the microRNAs and their expression levels of those expressed in at least 4 out 5 human neutrophil donors. The most abundant microRNA in all samples tested was miR-223, which negatively regulates granulocyte differentiation and fine tunes neutrophil function [38].

**Figure 2:**
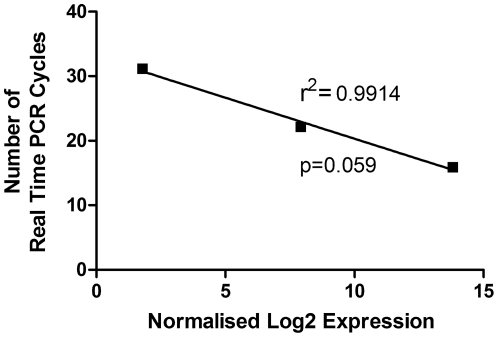
Good correlation is seen between microarray and real time PCR analysis of human neutrophil microRNA. The expression of 3 different microRNAs, miR-223, miR-29a and miR-486-5p were analysed by Real Time PCR and compared to the data obtained from the microarray. Real Time PCR and microarray analysis showed high correlation, r^2^ = 0.9914 (p = 0.059). Data are from 3 separate donors for Real Time PCR (purity 97%±0.58; apoptosis rates at 20 hours 70.87%±7.47) and 5 separate donors (purity 97.9%±0.21; apoptosis rates at 20 hours 62.4%±7.4) for the microarray.

To validate the microRNA expression levels determined by the microarray, real time PCR was performed on three further neutrophil donors (purity 97%±0.58; individual purities 97, 96, 98; 20 hrs apoptosis rate 70.87±7.47%) on selected microRNAs. Three microRNAs, miR-223, miR-29a and miR-486-5p were selected due to their different expression levels: high, medium and low/absent. The expression of these three microRNAs as determined by microarray correlated well with the number of PCR cycles required to reach the threshold, giving an r^2^ value of 0.9914, p = 0.059 (Figure 2).

### Neutrophils express several microRNA clusters

**Figure 3:**
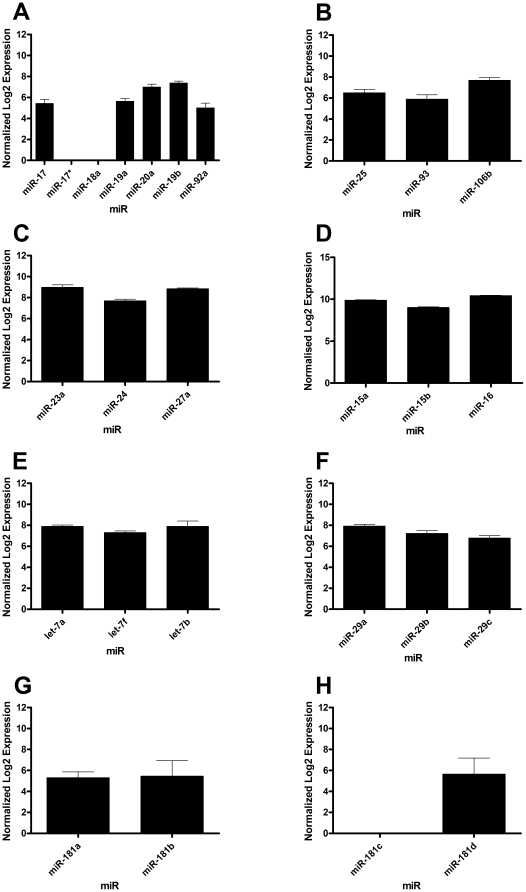
Freshly isolated human neutrophils express cotranscribed microRNA clusters. Total RNA from freshly isolated human neutrophils (purity 97.9%±0.21; apoptosis rates at 20 hours 62.4%±7.4; n = 5) were analysed for microRNA expression by microarray. Neutrophils were found to express 11 cotranscribed microRNA clusters. Expression levels are shown for the following clusters: A) miR-17-92, B) miR-106b-25, C) miR-23a-27a-24, D) miR-16-1 and miR-15b, E) let-7a-1 and let-7a-3, F) miR-29a and miR-29c, G) miR-181a and H) miR-181c.

Recent analysis of microRNA transcripts has revealed clustering, with many microRNAs being transcribed as a single precursor from a common promoter [39]. Many of these clusters have been associated with apoptosis and cell survival in cancer [39], therefore the microarray data were analysed for the expression of such clusters. We found human neutrophils expressed 11 out of the 25 clusters described by Yu *et al* [39]. The first such cluster to be analysed was the miR-17-92 cluster, which is located on chromosome 13 and contains seven mature microRNAs: miR-17 (17-5p), miR-17* (17-3p), miR-18a, miR-19a, miR-19b, miR-20a and miR-92a [40]. We found neutrophils expressed five members of this cluster (expressed in at least 4 out 5 donors), with the absent members being miR-18a and miR 17* (17-3p) (Figure 3a). The miR-17-92 cluster has 2 paralogs in mammals, the miR-106b-25 cluster which was found to present in human neutrophils (Figure 3b) and the miR-106a-363 cluster which was found to be absent. We also found the miR23a-27a-24 cluster, which has been reported to regulate both caspase-dependent and independent apoptosis [41] (Figure 3c). Two other clusters thought to be involved in the regulation of apoptosis are the miR-16-1 cluster and the miR-15b cluster. Both clusters contain the mature miR-16, but are transcribed from different locations within the genome. The miR-16-1 cluster is composed of miR-16 and miR-15a, while the miR-15b cluster is composed of miR-16 and miR-15b. All three mature microRNAs from these clusters were expressed in human neutrophils (Figure 3d). Further microRNA clusters found in human neutrophils were the let7a-1 and let7a-3 clusters (Figure 3e), the miR-29a and miR-29c clusters (Figure 3f), and the miR-181a cluster (Figure 3g). Interestingly, human neutrophils only express miR-181d and not miR-181c of the miR-181c cluster (Figure 3h).

### Regulation of neutrophil microRNAs

**Figure 4:**
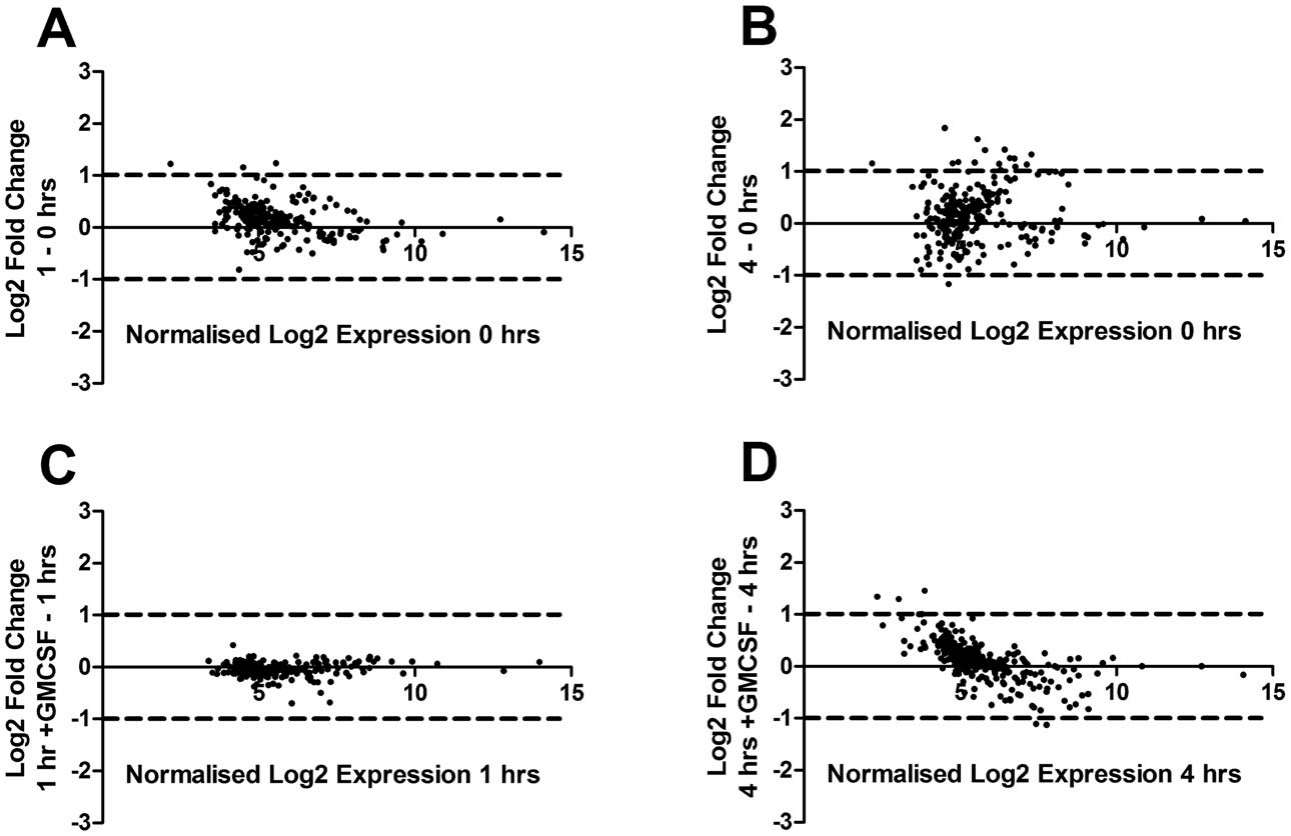
Regulation of neutrophil microRNAs. Total RNA from either freshly isolated neutrophils or those in culture for 1 or 4 hours with and without GMCSF was prepared (purity 97.8%±0.26; apoptosis rates at 20 hours 73.55%±3.39; with GMCSF 46.25%±3.19; n = 3) and were analysed for microRNA expression by microarray. A) MA plot showing regulation of neutrophil microRNAs after 1 hour in culture. B) MA plot showing the regulation of microRNAs after 4 hours in culture. C) MA plot showing the regulation of microRNAs after a 1 hour treatment with GMCSF. D) MA plot showing the regulation of microRNAs after a 4 hour treatment with GMCSF.

**Figure 5:**
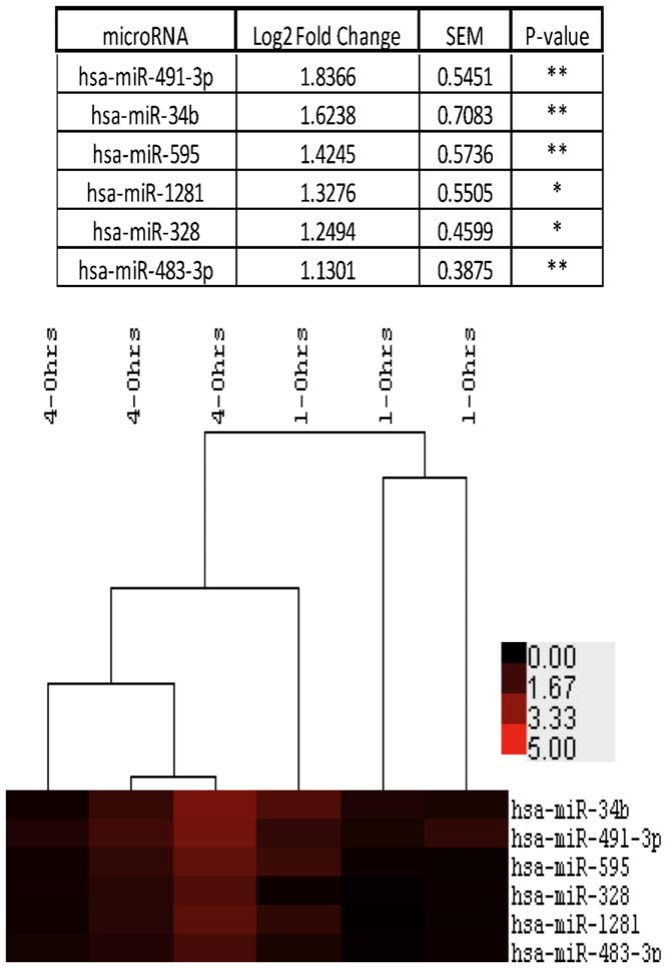
Neutrophil microRNAs are significantly upregulated after 4 hours in culture. Total RNA from either freshly isolated neutrophils or those in culture for 1 or 4 hours was prepared (purity 97.8%±0.26; apoptosis rates at 20 hours 73.55%±3.39; n = 3) and were analysed for microRNA expression by microarray. There were no microRNAs regulated after 1 hour in culture, however after 4 hours in culture miR-491-3p, miR-34b, miR-595, miR-328 miR-1281 and miR-483-3p were significantly upregulated, with none being downregulated. ** indicates p<0.01 and * indicates p<0.05 using the Friedman test with Dunn's multiple comparison. The heat map shows the regulation of the microRNAs in each individual donor at both 1 and 4 hrs.

Neutrophils are short-lived cells with a limited ability to regulate gene expression over time [34]. We investigated the regulation of neutrophil microRNAs over a period of 4 hrs in culture with and without GMCSF (50U/ml). At this time point negligible rates of apoptosis are seen, removing a potential confounding variable. Total RNA was prepared at 0, 1 and 4 hours after purification (Purity 97.8%±0.26; Individual purities 97.15, 98.25, 98; 20 hour apoptosis rate 73.55%±3.39, with GMCSF 46.25%±3.19 (n = 3)). To identify microRNAs that were regulated over time or upon treatment with GMCSF, only microRNAs that were expressed in at least 2 out of 3 donors at any one time point or treatment were selected for analysis. All expression values of Log(2)<1 were rounded up to 1 to remove any large fold changes when the expression values of the microRNAs were marginal. Figure 4 shows MA plots comparing normalised Log2 expression levels with the fold regulation, 1hr−0hrs (Figure 4a), 4hrs−0hrs (Figure 4b), 1hr+GMCSF−1hr (Figure 4c) and 4hrs+GMCSF−4hrs (Figure 4d). As might be expected, the majority of neutrophil microRNAs showed little or no regulation over time or upon treatment with GMCSF. To determine those microRNAs regulated in human neutrophils over time or upon treatment with GMCSF, data were ranked in order of fold change, and those with an average Log2 fold change of greater than 0.7 (1.62 fold) were selected for further analysis. In order to prevent undue influence of any single donor, only microRNAs that were regulated in each donor by greater than Log2 0.4 fold (1.32 fold) were selected, before statistical analysis of all 16 replicates for each donor using the Friedman test with Dunn's multiple comparison test. After 1 hour in culture, no neutrophil microRNAs were significantly regulated using this analysis. However, after 4 hours in culture, significant upregulation was seen for miR-491-3p, miR-34b, miR-595, miR-328, miR-1281 and miR-483-3p, with no microRNAs being significantly downregulated. Figure 5 shows the mean data for the regulation of these microRNAs, with the individual clustered donors below. Treatment of neutrophils with GMCSF did not result in the significant regulation of any microRNAs.

### Genomic location of regulated microRNAs and potential functional significance

Of the 6 microRNAs regulated during neutrophil culture, 3 are located within 3kb of known CpG islands (miRs-1281, 34b and 483-3p), and a further one (miR-328) is located within 10kb (CpG “shore”). These are areas of particular sensitivity to regulation by DNA methylation. This raises the possibility that epigenetic changes might regulate neutrophil function by changes in expression of microRNAs. Of further interest is the finding that 3/6 of the regulated microRNAs are within the introns of known protein coding genes. MiR-328 is in intron 12 on the opposite strand of the ELMO3 gene, miR-483-3p is within the 3′UTR on the opposite strand of IGF2, while miR-595 is in intron 1 of PTRN2. Interestingly, miR-153-2 is within intron 19 of PTRN2, but we were unable to detect this microRNA in our neutrophil samples. This raises the possibility that these microRNAs may be co-ordinately regulated with the genes in which introns they are located. However, microarray expression data is only available for two of these genes, PTRN2 and IGF2. Interestingly, PTRN2 is downregulated by the same conditions that cause upregulation of the intronic microRNA, and IGF2 was not detected in neutrophils. This suggests that post translational regulation of mature microRNA generation may be playing a part in the regulation of protein expression in human neutrophils. Once this mechanism is understood, this might prove a novel avenue for therapeutic manipulation of neutrophil function.

### Effects of microRNA regulation on neutrophil function

MicroRNAs can influence protein expression by changing either translation from existing mRNAs, or by influencing mRNA stability and hence transcript abundance. In order to determine the possible role of microRNAs in regulating the neutrophil transcriptome, we compared our data with a published microarray analysis of transcriptional profile of human neutrophils [34], which used similar culture conditions and timepoints to our study. By cross referencing changes in gene expression at mRNA level to our microRNA analysis we were able to identify regulated genes which were also predicted targets of regulated microRNAs. Of the transcripts downregulated in this analysis, 83 contained predicted binding sites for at least 2 of the 6 microRNAs upregulated after 4 hours (confirmed by at least 3 different algorithms, see Materials and Methods). This suggests that the observed changes in these transcripts may be caused by changes in microRNA abundance, or that changes in transcription and mRNA stability are co-regulated for these genes. In total, 176 of 602 down-regulated genes (29%) contained predicted binding sites for at least 1 out of the 6 microRNAs. Pathway analysis of the down-regulated genes with at least 2 predicted microRNA binding sites (http://david.abcc.ncifcrf.gov/tools.jsp [35]) revealed a significant enrichment (EASE Score p<0.05) of the following pathways: genes involved in the regulation of inflammation, mediated by chemokine and cytokine signalling; the Ras pathway; and regulation of the actin cytoskeleton (Table 1). Down-regulation of these pathways in tandem would reduce the ability of the neutrophil to respond to its environment – a key feature of senescence. There is also evidence of targeting of apoptotic pathways by microRNAs: BCL2L11 (BIM), BCLAF and PAK2 all contained binding sites for 4 out of the 6 microRNAs, although the significance of these remains to be determined. The potential for these microRNAs to target BIM is particularly intriguing given the recent observation that BIM protein is upregulated over time in neutrophils [42]. It is also interesting to note that the anti-apoptotic BCL-2 family member Mcl-1, which plays a significant role in regulating neutrophil lifespan, contained 1 binding site for miR-483-3p. However, no statistically significant regulation of microRNAs known to target Mcl-1 [28], [29] was identified.

**Table 1:** Regulated neutrophil microRNAs target multiple pathways, potentially downregulating neutrophil proinflammatory functions.

Inflammation mediated by chemokine and cytokine signalling pathways	
Gene	miR Binding Sites
PAK2	4
RHOB	2
PIK3CD	2
ITGAL	2
GNAQ	2
PTEN	2
NFATC3	2
CAMK2G	2
IL8RB	2

Ras pathway	
Gene	miR Binding Sites
PAK2	4
RHOB	2
PIK3CD	2
MAPKAPK	2
RS6KA3	2

Regulation of actin cytoskeleton	
Gene	miR Binding Sites
GNA13	2
PAK2	4
PIK3CD	2
VCL	3
ITGAL	2
MSN	2

Total RNA from either freshly isolated neutrophils or those in culture for 1 or 4 hours was prepared (purity 97.8%±0.26; apoptosis rates at 20 hours 73.55%±3.39; n = 3) and were analysed for microRNA expression by microarray. Genes potentially regulated by the upregulated microRNAs were downloaded from www.miRecords.com, and compared with those genes shown to be regulated over time. Genes within both datasets that were potentially regulated by at least 2 of the 6 regulated microRNAs were selected and pathway analysis performed. Pathway analysis of the downregulated genes with at least 2 predicted microRNA binding sites at http://david.abcc.ncifcrf.gov/tools.jsp [35], revealed a significant enrichment (EASE Score p<0.05) of genes involved regulation of inflammation mediated by chemokine and cytokine signalling, the Ras pathway, and the regulation of the actin cytoskeleton.

## Discussion

Neutrophils are short lived, terminally differentiated leukocytes that play a critical role in the destruction of invading bacteria and fungi. Many studies have reported that gene transcription and protein synthesis are key regulators of neutrophil function [16], [43]. MicroRNAs are a recently discovered small RNA species shown to regulate gene expression and may regulate the expression of up to 30% of all genes [26]. Many microRNAs have been linked to apoptosis in a variety of cell types and tumours [39]. We therefore sought to determine the basal expression of microRNAs in freshly-isolated human neutrophils, their regulation over time, and their regulation upon treatment with GMCSF, in order to identify novel regulators of neutrophil functional longevity.

We found highly purified human neutrophils express a distinct repertoire of microRNAs, with freshly isolated neutrophils (time 0) expressing 148 out the 851 human microRNAs (www.miRBase.com V12) in at least 4 out 5 donors on the Agilent Human V3 microRNA microarray. A recent similar array performed on human neutrophils after subjects had undertaken a bout of exercise found 282 microRNAs expressed by at least 50% of the samples in any one out of the two conditions [19]. Using a similar analysis method we found 261 microRNAs present in at least 2 out of the 3 samples at any one time point. The most abundant microRNA in all samples at any time point was miR-223, which has been shown to be highly expressed in mature neutrophils [38], [44] and to play a role in regulating neutrophil progenitor cell proliferation and function [38]. MiR-223 was not regulated in these experiments. Similar results were obtained in a study on microRNA expression in different hematopoietic lineages using and real-time PCR to determine the expression levels of 13 microRNAs [22]. Several other recent studies have also reported microRNA expression in human neutrophils using a variety of techniques [19], [20], [21], [45]. The next most abundant microRNA in human neutrophils was miR-720, of which very little is known and to date has no validated targets. Expression of miR-720 is not regulated over a wide variety of conditions, and it was identified by NormFinder as a suitable microRNA for normalisation of neutrophil data.

Neutrophils also expressed many microRNA clusters, in which several mature microRNAs are transcribed as a single precursor [39]. Many of these clusters have been shown to be dysregulated in many different cancers and to play a role in regulating apoptosis [46]. The first such cluster to be identified in our array was the miR-17-92 cluster, of which neutrophils expressed five out of the seven microRNAs. This is interesting as all seven are transcribed as one primary transcript, suggesting post-transcriptional regulation of this cluster within neutrophils or their precursors. We found that the absent members of this cluster were miR-17-3p (miR-17*) and miR-18a. The absence of both of these was not merely through non-detection using our custom microarray as studies on other cell types revealed both miR-18a and miR-17-3p using our custom array (personal communication, Craig Murdoch, University of Sheffield, UK). The exact function of miR-18a remains unknown, however one study using lentiviral mediated antagomir delivery into K562 cells found a positive role for miR-18a in cellular proliferation [47], possibly explaining its absence in terminally differentiated non proliferating neutrophils. The role of miR-17-3p remains unclear. The miR-17-92 cluster has two paralogs, the miR-106b-25 cluster which was found to be present in neutrophils and the miR-106a-363 cluster which was absent. Neutrophils also expressed high levels of the miR23a-27a-24 cluster which has been reported to be anti-apoptotic, with miR-27a targeting the activity of caspase-3 [48]. Other clusters found in neutrophils were the miR-16-1 cluster, the miR-15b cluster, the let-7a-1 and let 7a-3 clusters and the miR-29c and miR29a clusters. Many of the microRNAs within these clusters have been suggested to have a role in regulation of the cell cycle and apoptosis. Indeed over-expression of miR-15b results in cell cycle arrest in glioma cells [49], the miR-29 family targets Mcl-1 [28], and let-7a targets caspase-3 [50]. We also found that neutrophils expressed both members of the miR-181a cluster but only miR-181d of the miR-181c cluster.

To date there have been two previous studies on the regulation of microRNAs within human neutrophils, although none have studied neutrophil microRNA regulation over time or upon treatment with GMCSF. The first of these reported that miR-9 and miR-9* were upregulated in human neutrophils upon exposure to LPS [20]. We failed to detect miR-9 and miR-9* in any sample we arrayed, but we cannot rule out the possibility that the different techniques used (real time PCR vs microarray) could explain the differences seen. MiR-9 and miR-9* are highly expressed in monocytes [20]. Another more recent study used a similar array to that used in our experiments and identified 38 neutrophil microRNAs that were regulated by exercise [19].

There are currently only validated targets for one of the microRNAs found to be regulated, miR-328, which targets ABCG2 [51] and CD44 [52]. This makes defining an exact function of these microRNAs in neutrophils challenging. Similarly, siRNA and microRNA-mimic approaches in these fragile untransfectable cells are not possible with current technology. Development of a system for efficient miRNA/antagomir delivery into human neutrophils would have significant potential for delivering novel therapeutic and scientific advances. To address in part these shortcomings, we identified functionally valid targets for those microRNAs through the combination of predictive algorithms and reanalysis of previously published neutrophil microarray data [34]. Many of the genes that were downregulated over 3 or 6 hours in the microarray dataset were shown to have putative binding sites for the regulated microRNAs in their 3′ UTR. Pathway analysis of these genes revealed these microRNAs target pathways involved in the recruitment of neutrophils to sites of injury [53], [54]. This therefore suggests that microRNAs may regulate the ability of neutrophils to respond to chemotactic and proinflammatory stimuli, preventing such cells being recruited to the site of injury – key markers of senescence. It should be noted that the changes we observe in microRNAs at 4 hours are likely to be reflected in changes in protein levels and in function at significantly later timepoints.

We have shown that neutrophils express a selected repertoire of microRNAs and that a small number of these are regulated over time as neutrophils begin to undergo senescence/spontaneous apoptosis. Combining these data with neutrophil transcriptional profiles and microRNA binding site prediction algorithms has led us to identify microRNAs likely to play a role in the regulation of neutrophil senescence. The key targets of microRNAs in neutrophils remain to be identified; however it remains an intriguing possibility that therapeutic modulation of neutrophil function could be achieved through the modulation of microRNA pathways by microRNA mimics or antagomirs.

## Supporting Information

Table S1 **Freshly isolated human neutrophils express a selected repertoire of microRNAs.** Total RNA from freshly isolated human neutrophils (purity 97.9%±0.21; apoptosis rates at 20 hours 62.4%±7.4; n = 5) were analysed for microRNA expression by microarray. In total 148 microRNAs were found to be present (as indicated by the Feature Extraction software) in at least 4 out 5 donors. The data shown are the normalised Log2 values in descending order of abundance. MiR-223 was the most abundant in all samples.
(DOC)
