# Supplementary material for: Regulation of Neutrophil Senescence by MicroRNAs
Source: PLoS One. 2011 Jan 19;6(1):e15810. doi: 10.1371/journal.pone.0015810 (PMC3023715; doi:10.1371/journal.pone.0015810)
Supplement: Table S1 — Freshly isolated human neutrophils express a selected repertoire of microRNAs. Total RNA from freshly isolated human neutrophils (purity 97.9%±0.21; apoptosis rates at 20 hours 62.4%±7.4; n = 5) were analysed for microRNA expression by microarray. In total 148 microRNAs were found to be present (as indicated by the Feature Extraction software) in at least 4 out 5 donors. The data shown are the normalised Log2 values in descending order of abundance. MiR-223 was the most abundant in all samples. (DOC) [file pone.0015810.s001.doc]

| **microRNA** | **Mean** | **SEM** | **microRNA** | **Mean** | **SEM** |
| --- | --- | --- | --- | --- | --- |
| hsa-miR-223 | 13.8045 | 0.1650 | hsa-miR-188-5p | 5.8803 | 1.3604 |
| hsa-miR-720 | 12.2299 | 0.0446 | hsa-miR-1224-5p | 5.8679 | 1.3742 |
| hsa-miR-16 | 10.4234 | 0.0389 | hsa-miR-615-3p | 5.8447 | 1.2828 |
| hsa-miR-15a | 9.8637 | 0.0553 | hsa-miR-181d | 5.8421 | 1.3565 |
| hsa-miR-142-3p | 9.6822 | 0.7176 | hsa-miR-623 | 5.8097 | 1.3437 |
| hsa-miR-15b | 9.0216 | 0.0555 | hsa-miR-1300 | 5.7771 | 1.3190 |
| hsa-miR-23a | 8.9727 | 0.2405 | hsa-miR-1308 | 5.7588 | 1.3118 |
| hsa-miR-21 | 8.9113 | 0.1873 | hsa-miR-647 | 5.7563 | 1.2268 |
| hsa-miR-27a | 8.8305 | 0.0752 | hsa-miR-181b | 5.6526 | 1.3011 |
| hsa-miR-197 | 8.4142 | 0.7425 | hsa-miR-338-5p | 5.6495 | 1.3018 |
| hsa-miR-1260 | 8.3882 | 0.9394 | hsa-miR-19a | 5.6373 | 0.2420 |
| hsa-miR-26b | 8.0693 | 0.3030 | hsa-miR-539 | 5.6298 | 1.2747 |
| hsa-miR-574-3p | 8.0321 | 0.9124 | hsa-miR-140-3p | 5.6290 | 0.1186 |
| hsa-miR-29a | 7.9169 | 0.1697 | hsa-miR-765 | 5.5570 | 1.2740 |
| hsa-let-7a | 7.8998 | 0.1142 | hsa-miR-149 | 5.5282 | 1.1940 |
| hsa-let-7b | 7.8990 | 0.5006 | hsa-miR-664 | 5.5097 | 1.0559 |
| hsa-miR-574-5p | 7.8746 | 1.0430 | hsa-miR-610 | 5.5005 | 1.2716 |
| hsa-miR-24 | 7.6989 | 0.1481 | hsa-miR-221 | 5.4765 | 1.0067 |
| hsa-miR-106b | 7.6867 | 0.2560 | hsa-miR-122 | 5.4755 | 1.2632 |
| hsa-miR-32* | 7.5982 | 1.0991 | hsa-miR-1471 | 5.4593 | 1.2932 |
| hsa-miR-1281 | 7.5644 | 1.2774 | hsa-miR-425 | 5.4367 | 0.3063 |
| hsa-miR-26a | 7.4786 | 0.2623 | hsa-miR-17 | 5.4225 | 0.3850 |
| hsa-miR-483-3p | 7.4513 | 1.2478 | hsa-miR-195* | 5.4188 | 1.2217 |
| hsa-miR-1274b | 7.4314 | 1.1952 | hsa-miR-424 | 5.3962 | 0.2840 |
| hsa-miR-19b | 7.3775 | 0.1821 | hsa-miR-609 | 5.3765 | 1.1798 |
| hsa-miR-103 | 7.3395 | 0.1167 | hsa-miR-1538 | 5.3419 | 1.1981 |
| hsa-let-7f | 7.3056 | 0.1506 | hsa-miR-297 | 5.3292 | 1.2903 |
| hsa-miR-328 | 7.2248 | 1.4342 | hsa-miR-181a | 5.3100 | 0.5416 |
| hsa-miR-29b | 7.2179 | 0.2668 | hsa-miR-605 | 5.3093 | 1.1773 |
| hsa-miR-1825 | 7.1459 | 1.4014 | hsa-miR-1238 | 5.2893 | 1.1275 |
| hsa-miR-338-3p | 7.1284 | 0.2932 | hsa-let-7f-1* | 5.2797 | 1.1522 |
| hsa-miR-766 | 7.1008 | 1.3960 | hsa-miR-520d-3p | 5.2406 | 1.2261 |
| hsa-miR-142-5p | 7.0871 | 0.1635 | hsa-miR-629* | 5.2337 | 1.1542 |
| hsa-miR-107 | 7.0654 | 0.1473 | hsa-miR-640 | 5.1974 | 1.1657 |
| hsa-miR-885-5p | 7.0135 | 1.3558 | hsa-miR-1225-5p | 5.1972 | 1.1703 |
| hsa-miR-20a | 6.9970 | 0.2665 | hsa-miR-15a* | 5.1916 | 1.2025 |
| hsa-miR-1228* | 6.9914 | 1.5358 | hsa-miR-101 | 5.1709 | 0.2758 |
| hsa-let-7g | 6.9623 | 0.1238 | hsa-miR-382 | 5.1230 | 1.2437 |
| hsa-let-7d* | 6.8873 | 1.2467 | hsa-miR-1322 | 5.1012 | 1.1889 |
| hsa-miR-595 | 6.8722 | 1.5129 | hsa-miR-27b* | 5.0521 | 1.1900 |
| hsa-miR-29c | 6.7887 | 0.2176 | hsa-miR-34a | 5.0046 | 1.1876 |
| hsa-miR-923_v12.0 | 6.6880 | 0.1552 | hsa-miR-432* | 4.9989 | 1.1110 |
| hsa-miR-1909* | 6.6772 | 1.4728 | hsa-miR-92a | 4.9935 | 0.4610 |
| hsa-let-7i | 6.6757 | 0.4894 | hsa-miR-300 | 4.9808 | 1.1073 |
| hsa-miR-877* | 6.6567 | 1.4526 | hsa-miR-885-3p | 4.9717 | 1.3374 |
| hsa-miR-206 | 6.6200 | 1.4598 | hsa-miR-423-5p | 4.9684 | 1.1099 |
| hsa-miR-1268 | 6.6132 | 1.4928 | hsa-miR-30b | 4.9588 | 0.5835 |
| hsa-miR-140-5p | 6.6029 | 0.1812 | hsa-miR-346 | 4.9558 | 1.1403 |
| hsa-miR-1306 | 6.5471 | 1.4616 | hsa-miR-636 | 4.9458 | 1.1584 |
| hsa-miR-485-3p | 6.5371 | 1.4291 | hsa-let-7c | 4.9266 | 0.6216 |
| hsa-miR-25 | 6.4962 | 0.3166 | hsa-miR-200c* | 4.9216 | 1.1300 |
| hsa-miR-638 | 6.4883 | 0.6328 | hsa-miR-675* | 4.9184 | 1.0926 |
| hsa-miR-1280 | 6.4232 | 1.2798 | hsa-miR-1284 | 4.9025 | 1.0936 |
| hsa-miR-1207-5p | 6.3453 | 1.4384 | hsa-miR-548d-5p | 4.8987 | 1.1274 |
| hsa-miR-1180 | 6.3334 | 1.4272 | hsa-miR-194* | 4.8977 | 1.1479 |
| hsa-miR-1234 | 6.2314 | 0.8053 | hsa-miR-1225-3p | 4.8741 | 0.8578 |
| hsa-miR-1910 | 6.2182 | 1.3948 | hsa-miR-214 | 4.8717 | 1.1640 |
| hsa-miR-1224-3p | 6.1695 | 1.3488 | hsa-miR-550* | 4.8622 | 1.0831 |
| hsa-let-7d | 6.1591 | 0.2299 | hsa-miR-335* | 4.8490 | 1.0708 |
| hsa-miR-34b | 6.1123 | 1.0167 | hsa-miR-299-5p | 4.8356 | 1.0513 |
| hsa-let-7b* | 6.0769 | 1.3314 | hsa-miR-1539 | 4.8234 | 1.1120 |
| hsa-miR-1296 | 6.0622 | 1.3292 | hsa-miR-136 | 4.8084 | 1.0716 |
| hsa-miR-631 | 6.0426 | 1.3289 | hsa-miR-1236 | 4.7607 | 1.0387 |
| hsa-miR-324-3p | 6.0418 | 1.3068 | hsa-miR-16-2* | 4.6810 | 1.0733 |
| hsa-miR-940 | 6.0277 | 0.5963 | hsa-miR-29b-1* | 4.6157 | 1.1134 |
| hsa-miR-654-3p | 6.0147 | 1.2987 | hsa-miR-1229 | 4.6081 | 1.0441 |
| hsa-miR-22 | 5.9577 | 0.1638 | hsa-miR-331-3p | 4.5953 | 0.4401 |
| hsa-miR-432 | 5.9441 | 1.3709 | hsa-miR-19b-1* | 4.5475 | 1.0758 |
| hsa-miR-1915 | 5.9353 | 0.9295 | hsa-miR-320d | 4.5268 | 0.7193 |
| hsa-miR-937 | 5.9255 | 1.3003 | hsa-miR-1227 | 4.4532 | 0.9655 |
| hsa-miR-30c | 5.8955 | 0.6277 | hsa-let-7g* | 4.4187 | 1.0120 |
| hsa-miR-93 | 5.8866 | 0.4164 | hsa-miR-634 | 3.9516 | 0.8290 |
| hsa-miR-939 | 5.8828 | 1.3627 | hsa-miR-933 | 3.4516 | 0.8437 |
